# Supplementary figures and images for: Lowland tapir distribution and habitat loss in South America
Source: PeerJ. 2016 Sep 13;4:e2456. doi: 10.7717/peerj.2456 (PMC5028772; doi:10.7717/peerj.2456)

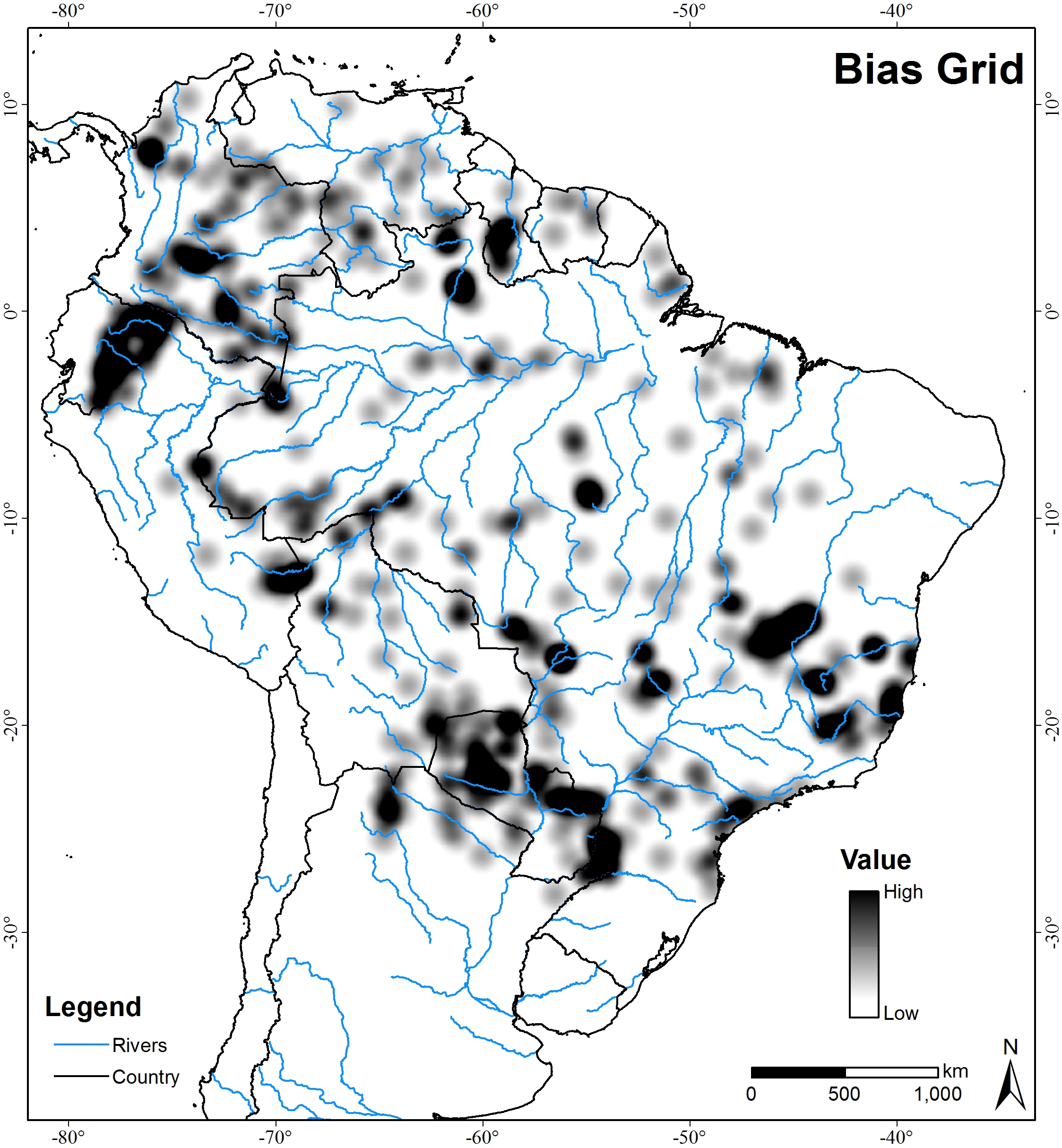

Supplement: Figure S1 — MAXENT bias grid, according procedures outlined by Elith, Kearney & Phillips (2010). The bias grid was used to down-weight the importance of presence records from areas with more intense sampling. The weighting surface was calculated based on the number of presence records within an area around any given cell (weighted by a Gaussian kernel with a standard deviation of 100 km). [file peerj-04-2456-s003.jpg]

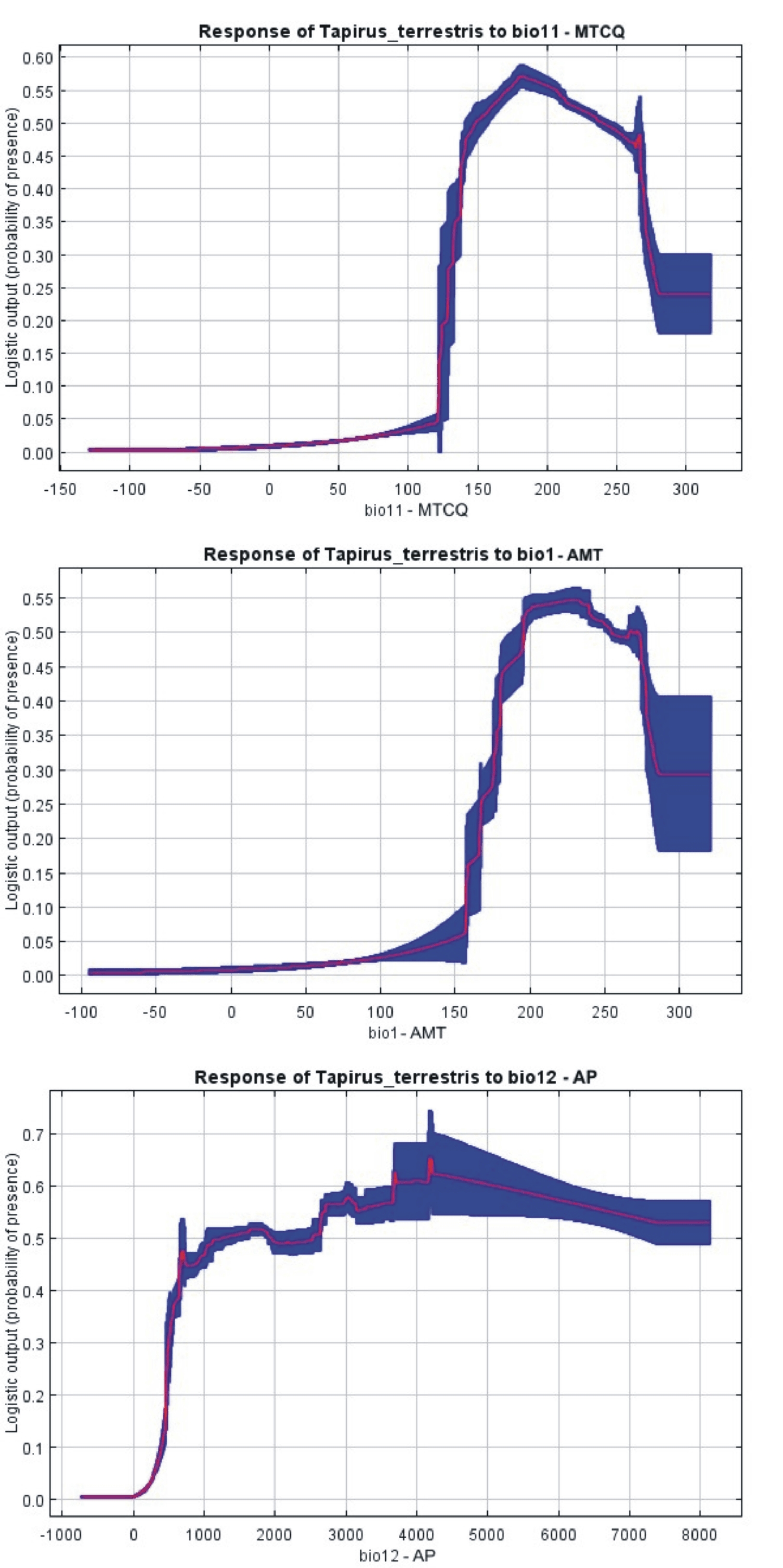

Supplement: Figure S2 — Response-curves of the variables in the MAXENT Tapirus terrestris distribution model. Mean Temperature of Coldest Quarter (MTCQ); Annual Mean Temperature (AMT); Annual Precipitation (AP). These curves show how each environmental variable affects the MAXENT prediction when all environmental variables are used to build the model. [file peerj-04-2456-s004.jpg]
